# NIDA-Drug Addiction Treatment Outcome Study (DATOS) Relapse as a Function of Spirituality/Religiosity

**DOI:** 10.17756/jrds.2015-007

**Published:** 2015-03-10

**Authors:** Stephen J. Schoenthaler, Kenneth Blum, Eric R. Braverman, John Giordano, Ben Thompson, Marlene Oscar-Berman, Rajendra D. Badgaiyan, Margaret A. Madigan, Kristina Dushaj, Mona Li, Zsolt Demotrovics, Roger L. Waite, Mark S. Gold

**Affiliations:** 1 Department of Sociology, California State University, Turlock, CA, USA; 2 Department of Psychiatry & McKnight Brain Institute, University of Florida College of Medicine, Gainesville, FL, USA; 3 Department of Neurology, PATH Foundation NY, New York, NY, USA; 4 Department of Clinical Research, National Institute for Holistic Addiction Studies, North Miami Beach, FL, USA; 5 Department of Psychiatry, Human Integrated Services Unit, University of Vermont Center for Clinical & Translational Science, University of Vermont College of Medicine, Burlington, VT, USA; 6 Department of Nutrigenomics, Igene, Inc., Austin, TX, USA; 7 Department of Addiction Research & Therapy, Malibu Beach Recovery Center, Malibu, CA, USA; 8 Dominion Diagnostics, LLC, North Kingston, RI, USA; 9 Departments of Psychiatry, Anatomy, & Neurobiology, Boston VA and Boston University School of Medicine, Boston, MA, USA; 10 Department of Psychiatry, University of Minnesota College of Medicine, Minneapolis, MN, USA; 11 Eotvos Lorand University, Institute of Psychology, Department of Clinical Psychology and Addiction, Izabella utca 46, H-1064, Budapest, Hungary; 12 Department of Nutrigenomics, RDSolutions, Inc., Salt Lake City, UT, USA; 13 Director of Research, Drug Enforcement Administration (DEA) Educational Foundation, Washington, D.C, USA; 14 Departments of Psychiatry & Behavioral Sciences at the Keck, University of Southern California, School of Medicine, CA, USA

**Keywords:** Relapse, Neurogentics, Reward Deficiency Syndrome (RDS), Spirituality, Genospirituality, Anomie, Social Bonds, Religion

## Abstract

**Background:**

The connection between religion/spirituality and deviance, like substance abuse, was first made by Durkheim who defined socially expected behaviors as norms. He explained that deviance is due in large part to their absence (called anomie), and concluded that spirituality lowers deviance by preserving norms and social bonds. Impairments in brain reward circuitry, as observed in Reward Deficiency Syndrome (RDS), may also result in deviance and as such we wondered if stronger belief in spirituality practice and religious belief could lower relapse from drugs of abuse.

**Methods:**

The NIDA Drug Addiction Treatment Outcome Study data set was used to examine post hoc relapse rates among 2,947 clients who were interviewed at 12 months after intake broken down by five spirituality measures.

**Results:**

Our main findings strongly indicate, that those with low spirituality have higher relapse rates and those with high spirituality have higher remission rates with crack use being the sole exception. We found significant differences in terms of cocaine, heroin, alcohol, and marijuana relapse as a function of strength of religious beliefs (x^2^ = 15.18, p = 0.028; logistic regression = 10.65, p = 0.006); frequency of attending religious services (x^2^ = 40.78, p < 0.0005; logistic regression = 30.45, p < 0.0005); frequency of reading religious books (x^2^ = 27.190, p < 0.0005; logistic regression = 17.31, p < 0.0005); frequency of watching religious programs (x^2^ = 19.02, p = 0.002; logistic regression = ns); and frequency of meditation/prayer (x^2^ = 11.33, p = 0.045; logistic regression = 9.650, p = 0.002). Across the five measures of spirituality, the spiritual participants reported between 7% and 21% less alcohol, cocaine, heroin, and marijuana use than the non-spiritual subjects. However, the crack users who reported that religion was not important reported significantly less crack use than the spiritual participants. The strongest association between remission and spirituality involves attending religious services weekly, the one marker of the five that involves the highest social interaction/social bonding consistent with Durkheim’s social bond theory.

**Conclusions:**

Stronger spiritual/religious beliefs and practices are directly associated with remission from abused drugs except crack. Much like the value of having a sponsor, for clients who abuse drugs, regular spiritual practice, particularly weekly attendance at the religious services of their choice is associated with significantly higher remission. These results demonstrate the clinically significant role of spirituality and the social bonds it creates in drug treatment programs.

## Introduction

Blum and associates were the first to explore the molecular biology and neurogenetic links to each step in the 12 steps programs adopted by many groups involved in recovery [1]. The importance of this present statistical analysis is to provide some evidence that acceptance of the 12-step doctrine, which contains a spiritual basis, may be linked to recovery from RDS behaviors. In the book, Blum’s group briefly discusses the development of the 12 steps and the co-founders struggles [1].

The role of neurotransmitters in the reward circuitry of the brain is well established and has been extensively reviewed elsewhere [2–6]. It is well known that *Homo sapiens* are biologically predisposed to drink, eat, reproduce, and desire pleasurable experiences. Impairment of the mechanisms involved in reward from natural processes leads to impulsive, compulsive, and addictive behaviors governed by genetic polymorphic-antecedents [2, 7, 8]. The genes include the DRD1 receptor; DAD2 receptor; DRD3 receptor; DAD4 receptor; DA transporter (DAT1) and the serotonergic 2A receptor (5-HTT2a). In addition, the serotonergic transporter (5HTTLPR); the catechol-O-methyltransferase (COMT), monoamine-oxidase and PER2 genes among others have polymorphisms that effect reward [9–11]. It is entirely possible that carrying reward gene polymorphisms may impact relapse.

The primary cause of drug-seeking behavior and the mechanism underlying a genetic predisposition to chronic drug use and relapse may be genetic polymorphisms or stress that induce a hypodopaminergic trait/state, regardless of the source [12]. Excessive liking/wanting of particular hedonic rewards might be the result of hypodopaminergic functioning and contribute to compensatory consumption for pleasure and to ameliorate RDS [13]. Dysfunction of the mesolimbic reward circuitry, essential for conserving a sense of well-being, results in drug abuse and relapse [14, 15]. Here we explore the link between the concept of spirituality/religiosity and relapse and recovery.

### Genes, religion, temperament and spirituality

“*Genospirituality*,” refers to the relationship of genetics to spirituality. That is; religious faith seems genuinely to lift the spirit, although it is hard to determine whether it is the God part or the community aspect that does the heavy lifting [16]. In terms of gene polymorphisms and associations to temperament, spirituality, God, and religion quite a few have been reported in the literature [17–24]. We are suggesting that while more in-depth work is required before we could simply accept this genetic notion that acceptance of a higher power is related to genetic antecedents, we do, however, point out this possibility.

Taking a spiritual inventory as part of clinical history is considered a valuable additional tool for medical treatment and diagnosis [25]. Clinical studies are beginning to clarify the contribution that spirituality and religion make to the coping strategies of many patients use in dealing with severe, chronic, and terminal conditions [26]. Interestingly, although twin studies of spirituality showed that the unique environment and genes each accounted for half of the variance, the shared environment including cultural influences accounted for none of the variance [27]. In contrast, shared environment including cultural transmission accounted for a significant percent of the variance in church attendance. Spirituality then, may be an intrinsic biological trait while religion is transmitted culturally, at least in part, from generation to generation [28–31].

Optimism would have positive selective value since it has been shown to promote a quicker recovery from disease and better health. Newberg et al. [32] suggested that the neurological machinery of spiritual transcendence may have evolved for mating and arisen from neural reward circuitry [the limbic system]. Thus, there is an association of spirituality with a “feel good” sensation. Comings [18–20] argued that “Spirituality” has to do with a feeling of connection with something greater than oneself that can include any form of social order.

Kendler and Myers [33] in discussing church attendance and genes pointed out individuals increasingly shape their social environment as they mature, somewhat based on their temperament that has been influenced by their genetic makeup. Familial and social environment influence the frequency of church attendance when individuals are young and living at home. These influences reduce levels of substance use. In contrast, during adulthood high levels of church attendance mainly reflect genetically influenced temperamental factors that are protective against substance use. Religious people like Jews, Muslims, Christians and others have been the subject of many investigators. According to Levin [34] debate about the role of genes amongst religious groups, involves both identity and religiosity and seems to frame who we are and our concept of a higher power.

### The social bonding process by which spirituality may lower deviance, crime, and substance abuse

Three seminal works concerning religion and deviance by Durkheim [35–37] offer clarity as to how spirituality sociologically influences remission can be found. Durkheim is considered the father of empirical sociology. He published that with their increased division of labor and rising social stratification, the advances of industrial societies had led to a crisis. He described this crisis as disorder, and anomie (meaning a state of “normlessness” in which there is a loss of “expectations” concerning what goals people should have in life and acceptable means to get there) [35]. Durkheim became a proponent of structural functionalism and noted that the major social institutions (religion, education, family, economy, and government) are found in every culture, without any exceptions. He concluded that each must be essential for cultural survival since no human cultures exist without them. In addition, the primary social function of religion is socialization that produces healthy norms, values, and social bonds within the division of labor [35]. He also published that deviance, such as suicide, was more closely linked to religion than education. In particular, he pointed out that Catholics were significantly less likely to commit suicide than Protestants due to much stronger norms and social bonds coming from their religious beliefs [36]. In 1912, he compared religious practices and beliefs in simple and complex cultures and found they were dependent on the other social institutions working together to protect society [37]. Durkheim stated, “… Religion is a unified system of beliefs and practices … which unites [society] into a single moral community… [among] all those who adhere to them” and that these social bonds lower crime, deviance, and addiction due to the social bonds that are created [38]. Activities such as praying together, reading religious works together, attending religious ceremonies together, or even watching religious activities together on television would provide social interaction. It follows that drug abuse might be lowered by spiritual activities that produce social interaction and bonds between group members such as 12-step membership.

### Defining difference between Spirituality and Religiosity

In an attempt, not to confuse the issue between the concepts related to Spirituality and Religiosity we point out that religion according to Durkheim [35] is a system of beliefs that unites people. Spirituality is a pattern of practices. In this article, we interchange religion and religiosity but refer to spirituality when it is related to a pattern of practices.

This is the first *posthoc* study (PUBMED search 8-1-13) to determine the role of spirituality and relapse utilizing the large Drug Abuse Treatment Outcome Study (DATOS) cohort developed by the National Institute on Drug Abuse (NIDA).

## Methods

### Subject participation

This article is a prospective study funded by the United States Department of Health and Human Services, the National Institutes of Health, and the National Institute on Drug Abuse between 1991 and 1994 (doi:10.3886/ICPSR02258.v5). It was designed to measure the outcomes of adult drug abuse treatment in 11 representative cities during 1991 and 1993. Outcome interviews were used at 1, 3, 6, and 12 months after entry among 10,019 clients. There were four types of programs: (a) outpatient methadone, (b) short-term inpatient, (c) long-term residential, and (d) outpatient residential. Twelve-month relapse and spirituality data were obtained for 2,947 of the 2,966 clients who were contacted. Nineteen clients declined to answer the spirituality questions.

### Subject demographics, pre-intervention drugs of choice, treatment modality, and the amount of relapse on each specific drug in DATOS

Our final sample was 64.5% male. Afro-American/blacks compromised the largest group at 46.8% followed by Caucasian/whites at 40.5% with Hispanics making up 10.5%. Crack and/or cocaine were the primary drug problem for 47.3% followed by heroin for 25.8% and alcohol for 9.9%. Treatment modalities were short-term inpatient (27.1%), outpatient drug free (25.7%) residential (22.7%), and methadone maintenance (24.4%). Only 33% were married or living with someone as married, and the mean age was 33.6 years with a mode of 31.

Table 1 rank-orders, the number of clients who reported relapse on specific drugs during treatment. Rank-order is important because it shows that over 90% of all drug use after entering treatment was concentrated in half the drugs that were abused. The concentration we found meant that the combined relapse index we used might mask any differential effects on the less frequently abused drugs, and results should not be generalized to all drugs without careful consideration of each alone.

### The Reliability of the self-report substance abuse among this population

A review of the DATOS literature suggests that these self-report data are quite reliable as only 8.8% of those who reported abstinence from cocaine/crack use, tested positive with biological assays [39]. The small positive result is crucial since the results are dependent on self-report data.

### Five main hypotheses

For the five main hypotheses, it is to be noted that each question simply provides the subject with five answers from which he/she selects one and as such there are no rating scales. The central hypothesis stated that clients with high spirituality would self-report significantly less drug relapse involving all drugs found in Table 1 combined, when compared to clients with low spirituality. The hypothesis was tested across five different ordinal measures of spirituality: (a) strength of religious beliefs, (b) frequency of attending religious services, (c) frequency of reading religious books, (d) frequency of watching religious programs, and (e) frequency of meditation/ prayer.

Each selected spirituality measure is consistent with Durkheim’s conclusions that all human cultures have spirituality that provides an essential function. Functionally, it reinforces culturally specific expected behaviors and values that produce social bonds and interaction with others resulting in inhibition of deviance, such as drug abuse. Theoretically, it follows that each of the five DATOS spirituality measures should be highly associated with remission. High spirituality in each measure is an indirect gauge of strong bonds to others with similar beliefs promoting conformity instead of deviance. It also follows that some measures such may produce more social bonds and less relapse than others. For example, as attending religious services would present more opportunities for social bonds than watching religious programs since the former must involve social interaction while this may or may not occur viewing a television program.

### Secondary hypotheses

Table 1 shows that, the distribution of relapse is heavily skewed toward a few drugs. Thus, we tested whether the relationship between spirituality and relapse held for each drug for which there existed sufficient relapse numbers individually. Each spirituality measure contains 4 or 5 categories. We reasoned that since significant clinical differences may be masked by the combined index we separated the psychostimulants into cocaine and crack to provide independent data. The dependent variable in these secondary analyzes was 12-month remission or relapse from any drug that more than 50 clients had used to relapse. The drugs were: (1) alcohol intoxication; (2) marijuana/hashish; (3) cocaine; (4) heroin; (5) crack; (6) narcotics and/or opiates; (7) depressants and/or downers and (8) benzodiazepines.

### Statistical analysis

The *post hoc* data was analyzed using both Pearson Chi- Square and Linear Association tests. It is noteworthy that the SPSS package that we utilized provides for regression results as well. The regression results are found in each table under the chi-square statistics and consistently match the level of significance we found using chi-square and we corrected for type one errors as well.

## Results

**Figure 1:**
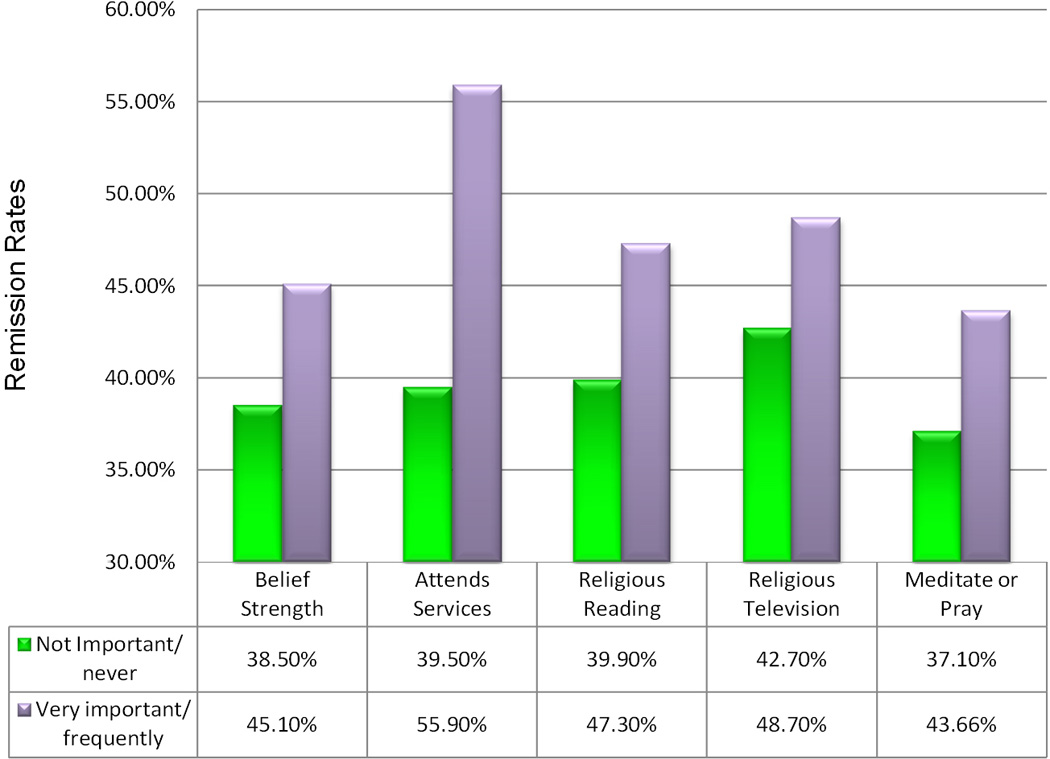
Remission rates associated with five different measures of spirituality

The results of the main hypothesis are presented in Tables 2 to 11. Each table (Tables 2,4,6,8,10) uses a different measure of spirituality that can be associated with the creation of social bonds, norms, and values that lower deviance from the norm, in this case, addiction (see Figure 1). Table 12 presents the results for more frequently abused drugs individually. The outcome variable of relapse or remission in each subtype includes relapse to all drug abuse or remission during the previous year.

The percentage who reported no drug abuse or alcoholic intoxication during the year after treatment entry; is broken down by no or very high spirituality. We found an increase in remission from all drugs combined when spirituality is very high as opposed to absent.

The main finding is that the percentage of the 2,947 subjects who achieved remission rises significantly as spirituality rises (p < 0.0005) across all five measures. In order to be identified as in remission in Tables 2 to 11 and Figure 1, a subject had to indicate that during the previous year, he or she had not used any drugs. The list included alcohol to the point of intoxication, marijuana, hashish, heroin, cocaine, crack, PCP, LSD. It also included narcotics, opiates, methadone, Dilaudid, downers or depressants, sedatives, barbiturates, benzodiazepines, tranquilizers, methamphetamines, inhalants, or other illegal drugs. Participants who were the highest in terms of spiritually consistently had higher remission rates than those without spirituality; they were also better than those with average-spirituality. The subjects who were the lowest in terms of spirituality had the highest relapse rates.

With regard to religious beliefs (see Tables 2 and 3) for those subjects who had the lowest belief, remission from all drugs of abuse during the past year was 38.5%. In contrast to those subjects who had the highest belief remission from all abusable drugs during the past year was 45.1%. The difference (see Table 3) was significant whereby the Pearson Chi-Square =15.178; df =3; p=0.028 and the Linear Association =10.650; df=1; p=0.006.

Tables 4 and 5 show that participants who attended religious services more than once a week had remission rates 16.4% higher than participants who did not attend religious services more than once a week. It is noteworthy that the strength of the association in Table 5 is stronger than the association found in Table 3 [Pearson Chi-Square =40.785; df=5; p<0.0005 and Linear Association = 30.453; df = 1; p<0.0005].

Tables 6 and 7 show that participants who reported reading religious books more than once a week also reported 14% more remission than participants who never read religious books; a significant association [Pearson Chi-Square = 27.190; df=5; p<0.0005 and Linear Association =17.309; df = 1; p<0.0005]. In addition, we also found significantly higher remission in participants that frequently viewed religious programs (see Table 8 and 9) during remission period (Pearson Chi-Square =19.024; df=5; p=0.002). Similarly, we found significant better remission in participants that frequently meditated/prayer during remission period (see Table 10) [Pearson Chi-Square =11.30; df= 5; p=0. 045 and Linear Association = 9.650; df=1; p=0. 002] (see Table 11).

The bottom row of Table 12 compares all drugs combined on each of the five measures of spirituality. It shows that those attending religious meetings most frequently produced the largest reduction in relapse 10.2% while the other four measures produced reductions between 6% and 7.4%. Attending religious meetings is the only one of the five measures that directly quantifies the social interaction and social bonds that Durkheim deemed to be so important for controlling deviance. However, the differences in remission as a function of spirituality were still statistically significant at the 0.0005 level for 11 drugs. They were alcohol intoxication, marijuana/hashish, heroin, cocaine, crack, narcotics and opiates, depressants and downers, benzodiazepines, and even sedatives, tranquilizers, and barbiturates. A few clients had relapsed on the latter three drugs. The results were not significant for eight drugs. The question of why relapse from methamphetamines, PCP, LSD, hallucinogens, inhalants, amphetamines, methadone, or Dilaudid, were not statistically significant remains unanswered. It could be that the number of relapses on those drugs was insufficient inferential testing, or there may be no relationship with these drugs. We suspect the former rather than the latter since relapse on these eight drugs only averaged 19.1 clients per drug, and that produces insufficient power. However, Table 12, produced other important findings including the fact that the association varies among drugs, but no result was more intriguing than the observation involving crack and cocaine. Both were significant at the 0.0005 level in opposite directions. Specifically, subjects with high spirituality produced 20.7% *less* relapse on cocaine than subjects with no spirituality while subjects with high spirituality produced 18.4% *more* relapse on crack that subjects with no spirituality.

A significant direct association was also found between higher spirituality and remission for some of the less popular drugs such as downers or depressants, sedatives, barbiturates, benzodiazepines, and other tranquilizers with p < 0.0005. There were six exceptions that were not significantly associated at even 0.05, perhaps due to comparatively infrequent relapse from these drugs among the DATOS clients. They included methamphetamines, inhalants, Dilaudid, methadone, PCP, and LSD. As sated above the regression analysis consistently match the level of significance.

### Limitations, Strengths and Suggestions

#### Design

The greatest limitation of all correlational studies is that they never can eliminate all rival explanations for the results. This study deign is insufficient to test for causation. Only a parallel randomized controlled trial can do so similar to pharmaceutical trials along the guidelines developed for CONSORT. However, correlational studies such as this often to lead to such trials.

#### Validity and reliability

Although we have no problems with the wording of the federal questions on spirituality, this field could be strengthened by measuring the correlation between them and alternative questions found in validated psychometric instruments and sociological surveys that might better tap into why such a strong association exists. This might lead to further support or rival explanations.

#### Statistics

It would be interesting to build measures that were true ratio scales instead of ordinal and test them against these questions to see if interval scales or at least dichotomous nominal ones could be created that would allow more sophisticated analyses above ordinal level measures.

#### Clinical ramifications

If a causative underlying mechanism was as simple as Durkheim and Cohen suggested social bonds to social institutions, there is a rich literature on how to use such knowledge in the addiction counseling literature. Motivational Interviewing followed by Cognitive Behavioral Therapy, Rational Emotive Therapy and others touch on building bridges and support that are different forms of social bonds.

Importantly, each of the five measures that we evaluated significantly correlated with each other, and as such we treated them separately. Since each is very significant standing alone, we decided not to combine the five measures into one unit. The reason for this decision is that the combination of measures would mask the individual-measures, and the individual-measures have implications for clinicians. For example, encouraging clients who believe to participate each week in their faith may lower relapse significantly.

A limitation of this study may have to do with the assumption that attendance at a religious activity encompasses social interaction. However, most sociologists would argue that (symbolic interaction) the dominant theoretical framework in sociology today would state that people construct their behaviors on the basis of their interpretation of others actions. People then adjust their constructed behaviors to be consistent with those that would receive the approval of others. Thus, a person who attends a religious service out of a desire to do so on a regular basis, need not speak to anyone there. They will, however, still interact non-verbally in a conversation of symbols via expressions on the face and body. Interaction can be, for example, verbal or symbolic including simple gestures, eye contact, smiles, hand movement, which have powerful effects on how participants interpret the meaning of what they observe.

In addition, we would like to point out that relapse in the literature is associated with the type of program, time-in treatment, and the severity of addiction as important variables. However, we did not attempt to control for them in this *posthoc* study. However, that does not bring our results into question. It would create an impossible standard for scientists-namely the scientist must control for every independent variable that affects the outcome variable, a standard that no scientist could ever meet.

Finally, we did report a very strong association between relapse and religious participation in which relapse is lower or higher by nearly 20% by this one factor alone. The limitation is that our design is a correlational study that is not capable of proving whether the results are “causative.”

## Discussion

Our overall results of a 42% reduction in indicators of drug use are consistent with what others have reported, i.e., “about 50” with ours being slightly more conservative due to the inclusion of all drug abuse in our operational definition. The literature notes significant differences in outcomes based on types of programs, length of time in treatment, and pre-admission severity, but as Hubbard et al. [39] point out, the previous findings “do not indicate who benefits the most from which treatment, and why.” This article suggests that those with higher spirituality are just as likely to remain in remission as clients who either were in treatment for shorter periods or had fewer severe problems before admission. While we did not address the role of family or others in our *posthoc* analysis we propose that support for spirituality should be considered as a useful tool in treating addiction by creating social bonds as well as any other practices that do so such as sponsors and family members who promote normative behavior.

Briefly, according to structural functionalism, social norms cluster into social roles that also clusters into role-sets that in turn cluster into five major institutions that each have critical roles in maintaining the survival of every human culture.

The five include:

1. economics (which makes and distributes goods and services that are essential);
2. family (which provides necessary socialization of the young and replacements for those who die);
3. education (which provides further essential socialization and training to operate the critical economic systems);
4. political (which protects societal survival from those who would conquer from outside and those who would disrupt from within); and
5. religion (that reinforces socialization of essential norms learned about the family and during education).

Thus, according to Durkheim [35], accepting spiritual norms will increase the probability of resisting deviance and as such possibly drug abuse relapse. Moreover, Sutherland [38] added “differential association theory,” that says; the time spent with people we are “intimate” with will greatly influence conformity or deviance based on their belief systems. Finally, Travis Hirschi [40] found that social bonds (“attachment”) were the best predictors of conformity or deviance in over 3000 teenagers. The five selected measures: reading, watching, attending services, adhering to the norm of praying and strong belief in religion, are consistent with the social theories of the above scholars.

Tables 2–11 show that participants who attended religious services more than once a week had remission rates 16.4% higher than participants who did not attend religious services more than once a week. It is noteworthy that the strength of the association in Table 4 is stronger than that in Table 2. We hypothesize that this could be due to the increase in support from participating with other people in religious services in addition to religious beliefs. In short, the presence of two bonds not one.

When we evaluated different drugs of abuse, some interesting results became obvious (see Table 12). As mentioned earlier the five measures of spirituality produce between 6% and 10% less reported drug use among the most spiritual when compared with the least spiritual and including all drugs. However, it also shows that the magnitude of use varies, amongst all of the commonly abused drugs. For example, avoidance of alcohol intoxication was between 6% and 17% higher among those who were highly spiritual compared to those who were not spiritual. The differences among heroin users were less, ranging between 5% and 10% with the most spirituality always producing the least abuse. The differences among marijuana use ranged between 3% and 9% with the most spiritual again producing the least abuse. We hypothesize that these differences may be due to a number of environmental influences. The use of alcohol is legal compared to heroin and marijuana in most U.S. states (excluding, for example, Colorado and Washington and now Alaska and Oregon, where marijuana is now legal for recreational use). Thus, we are proposing that people who strongly believe in God and go to church will be more inclined to use alcohol (legal) than heroin /marijuana (illegal) and spirituality is more important in their lives having influence on their deviant behavior.

In marked contrast, there was no significant change in cocaine/crack use between the least and most spiritual. Closer analysis of cocaine and crack use separately showed that cocaine use was significantly lower among those who were most spiritual, but crack use was significantly higher producing a combined result of no significant difference. Specifically, there is a very strong and significant association between spirituality and cocaine without crack that is even stronger than the association between intoxication and spirituality. However, the relationship between crack and spirituality is in the opposite direction and just as strong and significant too at the 0.0005 level.

Crack users tend to be economically at the bottom, more so than any other addicts. It is interesting that the inverse relationship occurred with crack with addicts who were in short-term residential centers rather than long-term, they were generally on public assistance, and they had relapse rates about double that of the cocaine users. This is consistent with the 1950s theory of Albert Cohen who said that the lives of some extreme poor are not socially disorganized (as advocated by most sociologists), but were reorganized around norms that were the exact opposite of what the rest of society says is right [41]. He said they had three characteristics, negativistic, non-utilitarian, and malicious. Being negativistic means that if their family, teachers, and others who are supposed to be significant in their lives believe in God, they take the opposite position and deem spirituality as a shame looking for social support from one or two other friends who share their mistrust of all institutions. The crack finding is consistent with his concept of being negativistic. Rather than be spiritual, they believe that there is no God and that everyone has to look out for themselves. In short, these hard-core deviants derive great strength from mistrust of everyone due to their cynical nature and fierce independence for religion, school, family, work, and government. They may have had at one time a developed belief in God and a particular religion, but that has been extinguished since they feel abandoned and their former upbringing believe in God has little impact on their remission rate and in fact it is inversely related. Their strength comes from tremendous autonomy, but their greatest limitation is the inability of trusting anyone even when opportunity presents itself, which not only explains the inverse relationship, but the high relapse rate.

Drug abuse relapse was identified as a function of educational attainment in another statistical analysis of the DATOS cohort [42]. This result is not surprising, and is in complete agreement with Hirschi [40] who indicated that the social bonds to the educational system are incredibly important in avoiding deviance. In addition, our results are in agreement with Rosmarin et al. [43] who concluded that belief in God, but not religious affiliation, were associated with better short-term psychiatric treatment outcomes for depression. The relationship with depression was mediated by belief in the credibility of treatment and expectations for treatment gains.

The findings of this study are consistent with the existing consensus that clients with substance abuse disorders produce better outcomes when they have sponsors when compared with those who do not have sponsors. Our study has pragmatic implications for those who work with substance abuse clients. Those clients who are religious would be well advised to participate in the religious services of their choice each week since that amount of participation is associated with significantly lower relapse, about 20%. In the treatment of agnostic crack users, it would be prudent to identify if possible, the person with whom they most closely identify that is not a substance abuser and attempt to involve them in assisting the client.

## Conclusion

Although limited due to being a *posthoc* analyses, the finding that a stronger belief in religiosity/spirituality, significantly, reduces relapse from drugs of abuse has clinical relevance. It supports the perspicacity of Twelve Step Programs and the ability of social bonds’ to remedy lack of social norms as defined by Emile Durkheim. We propose that impairments in brain reward circuitry, as observed in Reward Deficiency Syndrome (RDS), lead to deviance from the norm. Based on this research, stronger spirituality could lower relapse from drugs of abuse and should be supported during recovery.

## Figures and Tables

**Table 1:** The distribution of relapse among various drugs after entry to treatment.

Drugs used during 12 months after admission to treatment	n relapsing	Percent of total responding who report using this drug after admission (%)	Cumulative n	Percent Relapse Index (%)
Marijuana or Hashish	469	34	469	22
Alcohol Intoxication	391	47	880	40
Heroin	314	23	1194	56
Crack	299	61	1493	70
Cocaine	188	38	1681	78
Narcotics or Opiates	110	8	1791	83
Depressants or Downers	94	7	1885	88
Benzodiazepines	65	69	1950	91
Methamphetamines	41			
Sedatives	35			
Methadone	33			
Hallucinogens	23			
Dilaudid	22			
Tranquilizers	20			
LSD	15			
Amphetamines	13			
Barbiturates	11			
Totals	2,143			

**Table 2:** The association between strength of religious beliefs and drug remission.

How important are religious beliefs in your life?	Yes to remission of all drug abuse in past year n (%)	No to remission of all drug abuse in past year n (%)	Totals
Not at all important	87 (38.5)	105 (75.5)	226
Not too important	117 (37.0)	134 (66.3)	316
Fairly important	312 (38.2)	343 (66.7)	816
Very important	716 (45.1)	553 (63.0)	1589
**Aggregates**	**1232 (41.8)**	**1715 (58.2)**	**2947**

**Table 3:** The association between strength of religious beliefs and drug remission statistics.

Stat Type	Value	df	p
**Pearson Chi-Square**	15.178	3	0.028
**Linear Association**	10.65	1	0.006

**Table 4:** The association between frequency of attending religious services and drug remission.

How often do you attend religious services?	Yes to remission of all drug abuse in past year n (%)	No to remission of all drug abuse in past year (n)	Totals
Never	451 (39.0)	691 (60.5)	1142
Less than once a month	235 (35.5)	431 (64.7)	666
About once a month	100 (41.2)	143 (58.8)	243
2–3 times a month	109 (47.0)	123 (53.0)	232
Once a week	264 (49.7)	176 (50.3)	531
More than once a week	71 (55.9)	56 (44.1)	127
**Aggregates**	**1230 (41.8)**	**1711 (58.2)**	**2941**

**Table 5:** The association between frequency of attending religious services and drug remission statistics.

Stat Type	Value	df	p
**Pearson Chi-Square**	40.785	5	<0.0005
**Linear Association**	30.453	1	<0.0005

**Table 6:** The association between frequency of reading religious books and drug remission.

How often do you read religious books?	Yes to remission of all drug abuse in past year n (%)	No to remission of all drug abuse in past year n (%)	Totals
Never	341 (39.9)	513 (60.1)	854
Less than once a month	178 (34.2)	234 (72.0)	521
About once a month	121 (39.5)	185 (60.5)	306
2–3 times a month	104 (47.3)	116 (52.7)	220
Once a week	156 (44,8)	192 (55.2)	348
More than once a week	330 (47.3)	367 (52.7)	697
**Aggregates**	**1230 (41.8)**	**1716 (58.2)**	**2946**

**Table 7:** The association between frequency of reading religious books and drug remission statistics.

Stat Type	Value	df	p
**Pearson Chi-Square**	27.19	5	<0.0005
**Linear Association**	17.309	1	<0.0005

**Table 8:** The association between the frequency of watching religious programs and drug remission.

How often do you watch religious programs?	Yes to remission of all drug abuse in past year n (%)	No to remission of all drug abuse in past year n (%)	Totals
Never	577 (42.7)	774 (57.3)	1351
Less than once a month	141 (35.3)	258 (64.7)	399
About once a month	141 (34.6)	166 (64.7)	254
2–3 times a month	78 (41.7)	109 (58.3)	187
Once a week	209 (44.0)	266 (56.0)	475
More than once a week	134 (48.7)	141 (52.3)	275
**Aggregates**	**1227 (41.8)**	**1714 (58.2)**	**2941**

**Table 9:** The association between the frequency of watching religious programs and drug remission statistics.

Stat Type	Value	df	p
**Pearson Chi-Square**	19.024	5	0.002
**Linear Association**	2.533	1	<0.111

**Table 10:** The association between frequency of meditation/prayer and drug remission.

How often do you meditate or pray?	Yes to remission of all drug abuse in past year n (%)	No to remission of all drug abuse in past year n (%)	Totals
Never	109 (37.1)	185 (62.9)	294
Less than once a month	73 (33.6)	144 (66.4)	217
About once a month	49 (40.8)	71. (59.2)	120
2–3 times a month	59 (41.0)	553 (63.0)	144
Once a week	123 (41.3)	175 (58.7)	298
More than once a week	818. (43.6)	1057 (56.4)	1875
**Aggregates**	**1231 (41.1)**	**1717 (58.2)**	**2948**

**Table 11:** The association between frequency of meditation/prayer and drug remission statistics.

Stat Type	Value	df	p
**Pearson Chi-Square**	11.33	5	0.045
**Linear Association**	9.65	1	0.002

**Table 12:** The percent who reported no intoxication, nor use of heroin, marijuana, cocaine, or crack during the year after treatment entry, broken down by no or very high spirituality.

Drug Types	Religious importance		Frequency of prayer		Attendance of religious meetings		Reading of religious books		Watching religious programs	
	Not at all	Very	Never	>1× per week	Never	>1× per week	Never	>1× per week	Never	>1× per week
**Alcohol intoxication**	41.50%	56.70%	49.40%	55.70%	49.20%	57.00%	45.80%	62.60%	48.40%	61.40%
**n**	130	829	174	997	622	284	476	369	731	153
**Abstinence difference**	−0.152		−0.063		−0.078		−0.168		−0.13	
**11.8% mean increase in intoxication remission when spirituality is very high as opposed to absent**										
**Heroin**	80.10%	85.60%	76.20%	85.60%	79.70%	89.40%	80.70%	90.50%	83.10%	87.60%
**n**	226	1591	224	1876	1145	528	854	698	1353	274
**Abstinence difference**	−5.50%		−9.40%		−9.70%		−9.80%		−4.50%	
**7.8% mean increase in heroin remission when spirituality is very high as opposed to absent**										
**Cocaine**	37.50%	71.40%	47.60%	69.30%	59.00%	75.10%	54.30%	73.50%	57.60%	70.40%
**n**	72	573	103	668	405	173	289	260	736	98
**Abstinence difference**	−33.90%		−21.70%		−16.10%		−19.20%		−12.80%	
**20.7% mean increase in cocaine remission when spirituality is very high as opposed to absent**										
**Crack**	57.50%	37.90%	57.10%	37.50%	47.80%	29.10%	49.50%	32.80%	47.90%	32.30%
**n**	73	578	105	677	410	175	293	262	443	99
**Abstinence difference**	19.60%		19.60%		18.70%		16.70%		15.60%	
**18.4% mean decrease in crack remission when spirituality is very high as opposed to absent**										
**Marijuana**	68.10%	75%	69.40%	72.70%	70.00%	78.20%	70.00%	76.40%	70.20%	78.90%
**n**	226	1594	294	1882	1146	532	857	700	1358	275
**Abstinence difference**	−6.90%		−3.30%		−8.20%		−6.40%		−8.70%	
**6.7% mean increase in marijuana remission when spirituality is very high as opposed to absent**										
**All drugs combined**	38.50%	45.10%	37.10%	43.60%	39.50%	49.70%	39.90%	47.30%	42.70%	48.70%
**n**	226	1589	294	1875	1142	531	854	697	1351	275
**Abstinence difference**	−6.60%		−6.50%		**−10.20%**		−7.40%		6.00%	
**7.3% mean increase in all drug remission combined when spirituality is very high as opposed to absent**										
